# How we do it—the use of peritoneal patches for reconstruction of vena cava inferior and portal vein in hepatopancreatobiliary surgery

**DOI:** 10.1007/s00423-022-02662-x

**Published:** 2022-09-22

**Authors:** O. Radulova-Mauersberger, M. Distler, C. Riediger, J. Weitz, T. Welsch, J. Kirchberg

**Affiliations:** 1 Department of Visceral, Thoracic and Vascular Surgery, University Hospital Carl Gustav Carus, Technische Universität Dresden, Fetscherstrasse 74, 01307 Dresden, Germany; 2 National Center for Tumor Diseases (NCT/UCC), Dresden, Germany; 3 German Cancer Research Center (DKFZ), Heidelberg, Germany; 4 Faculty of Medicine and University Hospital Carl Gustav Carus, Technische Universität Dresden, Dresden, Germany; 5 Helmholtz-Zentrum Dresden - Rossendorf (HZDR), Dresden, Germany

**Keywords:** Parietal peritoneum patches (PPP), Vascular resection, Liver surgery, Pancreatic surgery, Vascular reconstruction

## Abstract

**Purpose:**

Extended resections in hepatopancreatobiliary (HPB) surgery frequently require vascular resection to obtain tumor clearance. The use of alloplastic grafts may increase postoperative morbidity due to septic or thrombotic complications. The use of suitable autologous venous interponates (internal jugular vein, great saphenous vein) is frequently associated with additional incisions. The aim of this study was to report on our experience with venous reconstruction using the introperative easily available parietal peritoneum, focusing on key technical aspects.

**Methods:**

All patients who underwent HPB resections with venous reconstruction using peritoneal patches at our department between January 2017 and November 2021 were included in this retrospective analysis with median follow-up of 2 months (IQR: 1–8 months). We focused on technical aspects of the procedure and evaluated vascular patency and perioperative morbidity.

**Results:**

Parietal peritoneum patches (PPPs) were applied for reconstruction of the inferior vena cava (IVC) (13 patients) and portal vein (PV) (4 patients) during major hepatic (*n* = 14) or pancreatic (*n* = 2) resections. There were no cases of postoperative bleeding due to anastomotic leakage. Following PV reconstruction, two patients showed postoperative vascular stenosis after severe pancreatitis with postoperative pancreatic fistula and bile leakage, respectively. In patients with reconstruction of the IVC, no relevant perioperative vascular complications occurred.

**Conclusions:**

The use of a peritoneal patch for reconstruction of the IVC in HPB surgery is a feasible, effective, and low-cost alternative to alloplastic, xenogenous, or venous grafts. The graft can be easily harvested and tailored to the required size. More evidence is still needed to confirm the safety of this procedure for the portal vein regarding long-term results.

## Introduction

Multivisceral tumor resections are technically demanding procedures. However, they are the only curative option for patients with locally advanced malignant tumors offering potential long-term survival [1, 2]. The main goal is to achieve tumor clearance by means of a safe surgical procedure. Due to increasing experience in the last decades, vascular resection is now a routine part of extended hepatopancreatobiliary procedures in high-volume centers [3]. A range of surgical techniques (primary anastomosis, graft interposition, or application of patches) using a variety of autologous, xenogenous, or alloplastic grafts and patches are available for reconstruction. Maintaining vascular patency after reconstruction and achieving long-term recurrence-free survival after radical resection are essential for recovery and patient survival. Hence, autologous material is preferred to minimize the risk of infection caused by common septic postoperative complications like biliary leakage, postoperative pancreatic fistula (POPF), and postpancreatectomy acute pancreatitis (PPAP) after HPB procedures [4]. Further advantages of autologous grafts compared to alloplastic grafts include lower costs, no need for anticoagulation after reconstruction, and the option to tailor graft size exactly to the individual geometric need without limitations in size or shape.

In this study, we focus on the peritoneum as autologous tissue graft available for reconstruction of the IVC and PV. Given the first encouraging results in the literature, we adopted this technique and used peritoneal patches for vascular reconstruction in complex multivisceral tumor resections. The largest patient cohort reported to date (52 patients) showed peritoneal patches to be a feasible option for venous reconstruction in HPB surgery [5, 6]. Nevertheless, data in the literature are still very scarce and limited to case reports and small patient collectives mainly with liver resections [5].

We report our experience of using PPPs for venous reconstruction of the IVC and PV in liver and pancreatic surgical procedures and highlight important technical aspects.

## Patients and methods

Data were collected retrospectively from all patients who underwent reconstruction of the IVC and PV at our institution from January 2017 to November 2021. We found in total 59 cases with complex surgical multivisceral procedures where a patch or tube interponat for a vascular venous reconstruction was performed. Patients with arterial reconstructions and those with a direct suture vascular anastomosis were excluded since they have not been addressed in the research question. Fifty-eight of the patients included underwent planned elective surgery and in one case, a patch plastic with bovine pericard after thrombectomy of the external iliacal vein in case of graft necrosis after renal transplantation was performed.

We performed in total 48 complex reconstructions of IVC (*n* = 41) and the PV (*n* = 8) where a vascular interposition (tube or a patch) was used. Ringed synthetic graft tube (GoreTex®) was applied in 13 cases, when a caval segment has been resected; bovine pericardium (XenoSure® Biologic patch and LeMaitre Vascular®, USA), when a patch reconstruction was needed in 11 cases; an autologous vein in 9 patients for patch reconstruction and tube interposition; and PPPs were used in 17 cases.

We focused on liver and pancreatic resections with PPP vascular reconstruction, as these are procedures associated with significantly higher bacterial contamination and increased risk for septic postoperative complications [6]. Patients who received peritoneal patches for the reconstruction of iliac veins were excluded. In the short-term follow-up, data to assess vascular patency was collected from contrast-enhanced computed tomography (CT) scans with venous phase. If patients were asymptomatic, we did not perform a scheduled imaging. CT scan was performed in cases of postoperative complications or as a staging diagnostic during oncological follow-up. Imaging intervals were variable among the patient collective as a result.

Treatment was discussed in an interdisciplinary tumor conference preoperatively. The indication for resection was a malignant tumor in all cases and an extended oncological (R0) resection was performed.

Postoperative morbidity was evaluated based on the Clavien–Dindo classification of surgical complications [7]. The results were represented as median and interquartile range (IQR). For postoperative patency, we defined a complete occlusion if an imaging vascular contrast was absent and stenosis if half or less of the vascular lumen was contrasted associated with clinical signs (ascites, intestinal venous congestion, lower extremities edema). Thrombosis was diagnosed if a venous thrombus was found intraluminally in CT or ultrasound.

### Procedure and important technical aspects

**Fig. 1:**
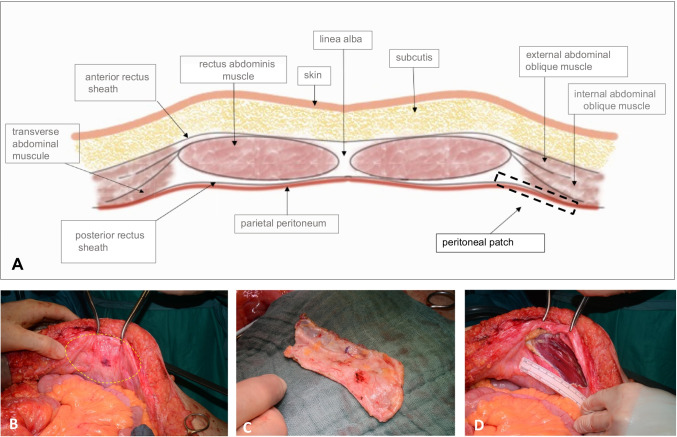
**A** Structure of the parietal peritoneum patch (PPP), **B** site of harvesting, **C** PPP, and **D** defect of the abdominal wall after PPP excision

**Fig. 2:**
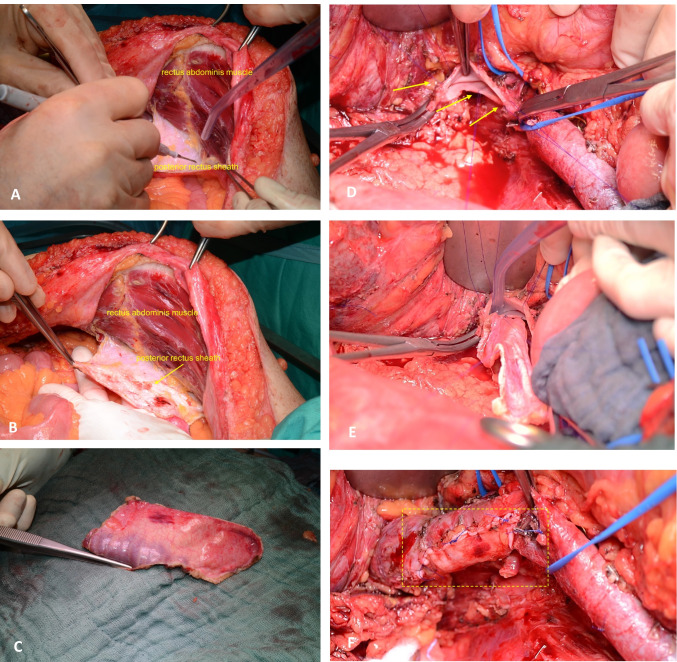
Surgical steps of PPP preparation. **A** Harvesting PPP from the hypochondrium. **B** and **C** Preparation is carried out in the layer between the rectus abdominis muscle and its posterior sheath. **D** The IVC is clamped proximally and distally to the tumor or longitudinally. Single traction sutures are placed at the patch and vessel corners to prevent twisting—arrows marked. **E** The patch is applied with the peritoneal surface on the luminal side of the vessel. It is important to ensure that the suture is tight but not strained in order to avoid stenosis of the vessel. **F** The clamp is removed, blood flow is restored and the suture is checked for sufficiency

Intraoperatively, the patients were placed in a supine position with the right arm abducted. Laparotomy was performed using a midline or transverse abdominal incision and reversed L-shaped incision for hepatic resection. After excluding metastatic spreading in the abdominal cavity, dissection was started based on tumor location. In most cases, vascular resection was one of the last surgical steps before tumor removal in order to obtain the best possible bleeding control and avoid prolonged interruption of blood flow. The exact degree of vascular involvement of the tumor was assessed intraoperatively. A PPP was needed if the defect of the vessel wall was less than 30% of its circumference; otherwise, a tube graft was preferred. The IVC or PV was first dissected and prepared for clamping at both sides of tumor infiltration. PPPs were harvested from an area of the abdominal wall with intact peritoneum before clamping the vein (Figs. 1 and 2).

PPPs were harvested from the right or left hypochondrium, the diaphragm, or from the posterior rectus muscle sheath with a peritoneum layer (Fig. 1). We prefer PPP from the right or left hypochondrium lateral to the rectus abdominis muscule for PV reconstruction (under consideration of the small presented patient number in the study), since the peritoneum is slightly thinner here compared to the ventral abdominal wall. In contrast, we prefer the thicker peritoneum with the posterior rectus layer of the ventral abdominal wall for vascular reconstruction of the VCI.

It is important not to harvest a PPP too small to bridge the vessel defect, in order to avoid a constricted vessel reconstruction that predisposes for thrombosis due to reduced local blood flow. PPP size was documented for eight patients after harvesting and before insertion of the patches. An important technical issue for the reconstruction is the size of the PPP, which is usually harvested larger than the defect of the vascular wall and a size reduction is performed before insertion to avoid PPP enlargement over time. In our institution, reconstruction was performed in a standard manner as a lateral patch plastic of the IVC or PV.

The surrounding fat tissue was left on the patch surface and the grafts were placed in saline solution (0.9% NaCl) until needed for the reconstruction (Fig. 1). The vein was side- or cross-clamped proximal and distal to the tumor. No systemic anticoagulation was administrated before or during clamping. After resecting the tumor, the vein defect was reconstructed by inserting the peritoneal patch with a running 4–0 (IVC) or 5–0 (PV) Prolene® suture. The patches were applied with the peritoneal surface on the luminal side of the vessel. As with direct venous anastomosis without patch/ graft interposition, it is important to loosely adapt the suture to avoid tension and create slightly slack knots to prevent stricture of the vein. Before final closure and restoring blood flow, retrograde and antegrade flushing was performed and local application of a heparin/saline solution (5000 IU heparin/500 ml saline) was used. After completing the suture, the clamps were removed to confirm sufficiency of the anastomosis (Fig. 2).

**Fig. 3:**
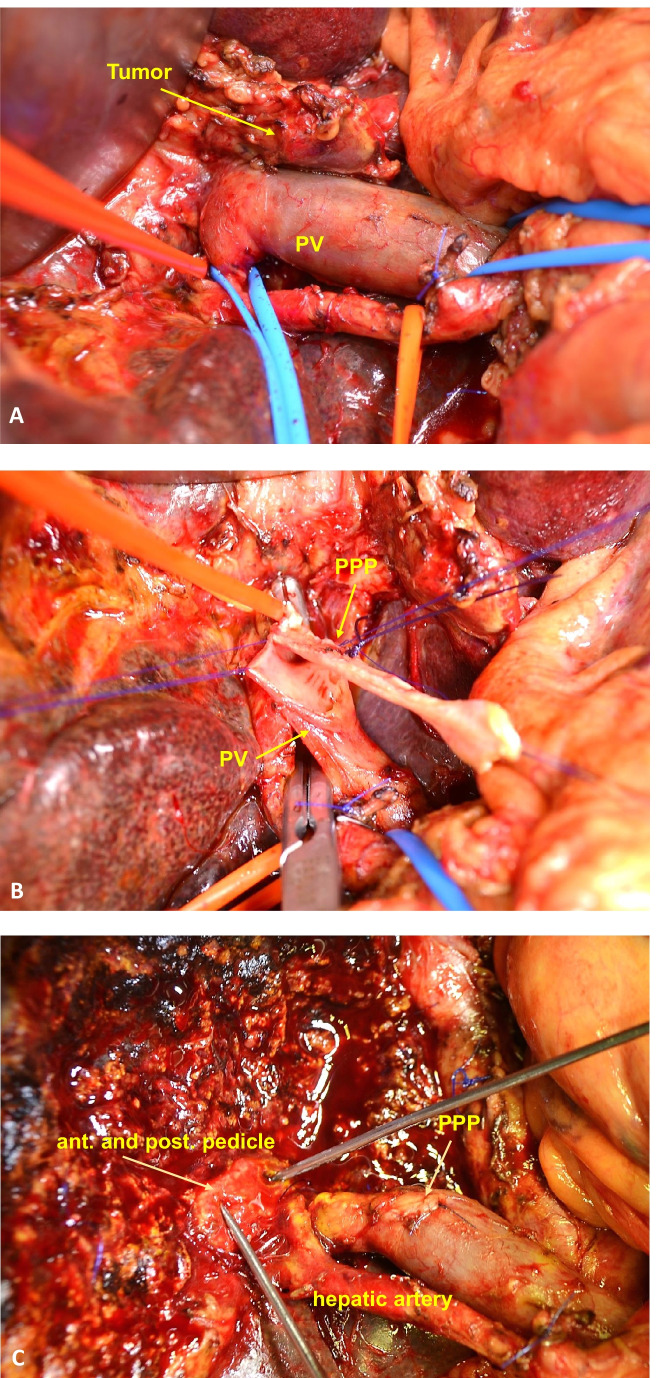
Reconstruction of the PV with PPP. **A** CCC infiltrating the portal vein; **B** reconstruction of the PV with PPP inserted by using a continuous 5–0 Prolene® monofilament suture; **C** liver parenchyma transection after PPP implementation

**Fig. 4:**
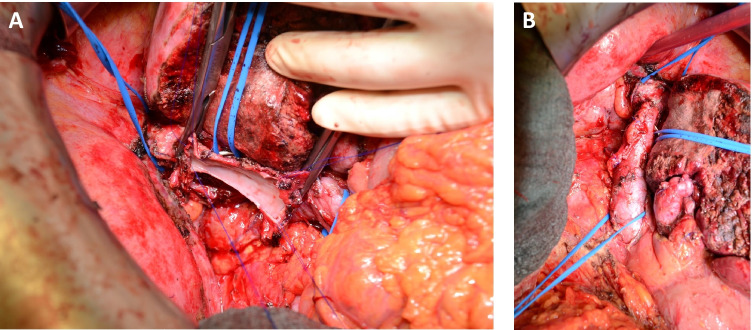
Reconstruction of the IVC with PPP. Patient with hepatocellular carcinoma infiltrating the IVC and the diaphragm. Resection was performed after transarterial chemoembolisation (TACE) of segments VII and VIII and portal vein embolization of the right portal vein. Right hemihepatectomy with tangential resection of the IVC and partial diaphragmatic resection achieved R0 removal of the tumor, and vascular reconstruction was performed with a PPP. The patient had a complication-free course apart from a superficial SSI and was discharged 12 days postoperatively. **A** IVC reconstruction with PPP. The patch is inserted by using a continuous 4–0 Prolene® monofilament suture. **B** After removing the clamp and restoring the blood flow, the suture is checked for bleeding and stenosis

**Fig. 5:**
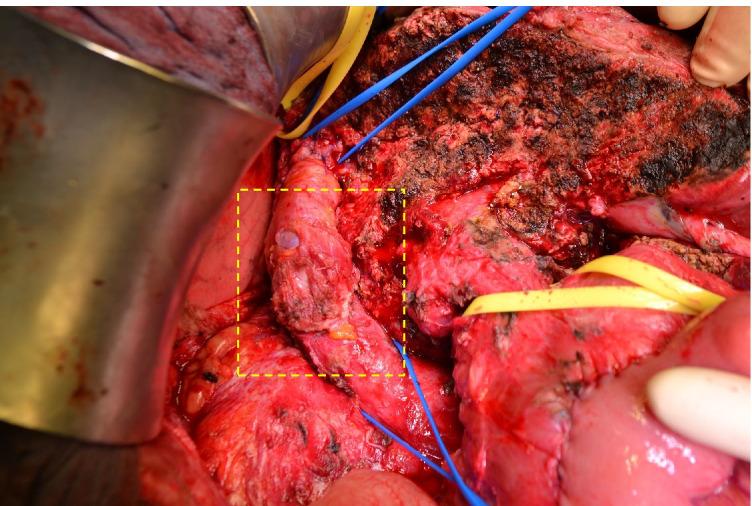
IVC reconstruction with PPP after resection of liver segment 4 for recurrent perivascular epitheloid cell tumor (PECom) infiltrating IVC. The patient had prolonged hospital stay of 17 PODs due to bilioma and pleural effusion. The patency of VC postoperative was not altered in CT

The surgical approach in reconstruction of PV (Fig. 3) and IVC (Figs. 4 and 5) did not differ. If possible, parenchymal hepatic transection was performed before vascular resection for liver procedures in order to avoid damaging the vascular reconstruction (Fig. 3). All patients received pharmacological prophylaxis with heparin (low-molecular-weight LMWH or unfractionated UFH) postoperatively. Treatment followed the German interdisciplinary, evidence- and consensus-based (S3) clinical practice guideline on venous thromboembolism prophylaxis based on patient-related risk factors and type of surgery [8].

## Results

Patients had a median age of 60 years (IQR: 53–71). Nine underwent hemihepatectomies, four had segmental liver resections, two had pancreatic resections, one underwent resection for paraaortic lymph node metastases, and one hepatic duct resection for bile duct carcinoma (Tables 1 and 2). In eight evaluable cases, PPP size varied between 150 and 3600 mm^2^. The exact patch size was not measured and reported in the other cases. Size was recorded after harvesting and before insertion of the PPP. No adverse events occurred in any of the patients during the operation. The median operating time was 413.7 min (IQR: 311.5–511) and median blood loss was 1205.8 ml (IQR: 650–1650).

**Table 1:** Patient characteristics

Patient	Age	Sex	BMI	ASA	Diagnosis	Surgical procedure	Blood loss	Site of reconstruction	Site of harvesting	Patch size mm^2^
1	66	f	24.6	II	Metastatic paraaortic lymph nodes of gastric carcinoma	Lymphadenectomy	350	VC	Hypochondrium	NA
2	60	m	29.2	III	CRLM	Left hemihepatectomy	2600	VC	Hypochondrium	NA
3	55	m	26.8	II	CCC	Right hemihepatectomy	2000	VC	Hypochondrium	1000
4	74	f	26	III	CCC	Left hemihepatectomy	1200	PV	Hypochondrium	600
5	71	m	23	III	CCC	Left hemihepatectomy with resection of the portal vein	1800	PV	Hypochondrium	NA
6	52	f	34.4	II	Gallbladder carcinoma	Right hemihepatectomy	700	VC	Hypochondrium	NA
7	49	f	25.3	II	CRLM	Right hemihepatectomy	500	VC	Diaphragm	800
8	67	f	24.2	II	Pancreatic carcinoma	PPPD with resection of the portal vein and vena cava	800	VC	Hypochondrium	NA
9	75	m	20.3	III	CCC	Right hemihepatectomy	3100	VC	Hypochondrium	NA
10	73	m	24.6	III	CCC	Hepatic duct resection	1100	PV	Hypochondrium	150
11	60	f	21.3	III	Pecoma of the liver	Liver segment resection	1000	VC	Hypochondrium	1600
12	53	m	22.1	II	Adrenal metastases of esophageal carcinoma	Liver segment resection	750	VC	Hypochondrium	1600
13	75	m	25	III	CRLM	Liver segment resection	800	VC	Hypochondrium	800
14	46	f	25	II	CRLM	Right hemihepatectomy	600	VC	Hypochondrium	NA
15	45	f	17.8	III	CRLM	Liver segment resection	1500	VC	Hypochondrium	NA
16	57	m	25	II	Pancreatic carcinoma	Distal pancreatic resection	400	PV	Hypochondrium	NA
17	63	m	31.7	II	HCC	Right hemihepatectomy	1300	VC	Hypochondrium	3600

*Abbreviations: CRLM*, colorectal liver metastases; *HCC*, hepatocellular carcinoma; *CCC*, cholangiocellular carcinoma; *CV*, caval vein; *PV*, portal vein; *NA*, not available

**Table 2:** Postoperative outcome

Patient no	Obstruction	ICU (days)	Hospital stay (days)	Postop. bleeding	POPF (P)/bilioma (B)	BDA leakage	CDC classification	30-day mortality	Follow-up (months)
1	No	1	8	No	No	No	3a	0	48
2	No	2	8	No	No	No	0	0	43
3	No	6	10	No	No	No	0	0	31
4	No	12	14	Yes	B	Yes	5	1	3
5	Yes	2	22	No	No	Yes	3a	0	18
6	No	44	44	Yes	B	No	4	0	1.5
7	No	3	33	No	B	No	3a	0	19
8	No	1	16	No	No	No	0	0	7
9	No	30	83	No	B	Yes	3a	0	1
10	No	4	14	No	No	No	0	0	11
11	No	5	17	No	B	No	3a	0	12
12	No	1	9	No	No	No	0	0	14
13	No	1	12	No	No	NA	0	0	6
14	No	3	15	No	No	No	1	0	14
15	No	2	7	No	No	No	0	0	13
16	Yes	112	131	Yes	P	No	4b	0	15
17	No	50	50	Yes	B	No	4b	0	1.5
Results	**12%**	**Median = 16.4 (IQR: 1.5–21)**	**Median = 29.2 (IQR: 10.5–38.5)**	**24%**	**n = 1 POPF/n = 6 bilioma**	**n = 3**	**CDC** ≥ **3b 24%**	**6%**	**Median = 17.1 (IQR: 6.5–19)**

*Abbreviations: ICU*, intensive care unit; *POPF*, postoperative pancreatic fistula; *CDC*, Clavien–Dindo classification [8]

We did not perform any revisions of the patches. One primary abdomen apertum due to intestinal edema with a planned “second look” relaparotomy was performed. Clinically relevant (CDC ≥ 3b) events with prolonged hospital stay and need of intervention occurred in four (24%) of the patients [7]. The main complications in these cases were POPF (*n* = 1), bilioma (*n* = 6), biliodigestive anastomosis leakage (*n* = 3), and postoperative hemorrhage (*n* = 4) due to impaired coagulation or vascular arrosion in pancreatitis.

The 30-day mortality rate was 6% (n = 1). One patient died from multiorgan failure as a consequence of a biliodigestive anastomosis leakage and sepsis. There were no immediate postoperative complications related to the peritoneal patch reconstruction.

Computed tomography images were available for evaluation of the vascular graft patency in 16 of the 17 patients at a mean follow-up of 17 months (1.5–48 months) after resection. We obtained information for the one patient who was lost for follow-up from his oncologist. He had no clinical signs of recurrence or venous obstruction.

**Fig. 6:**
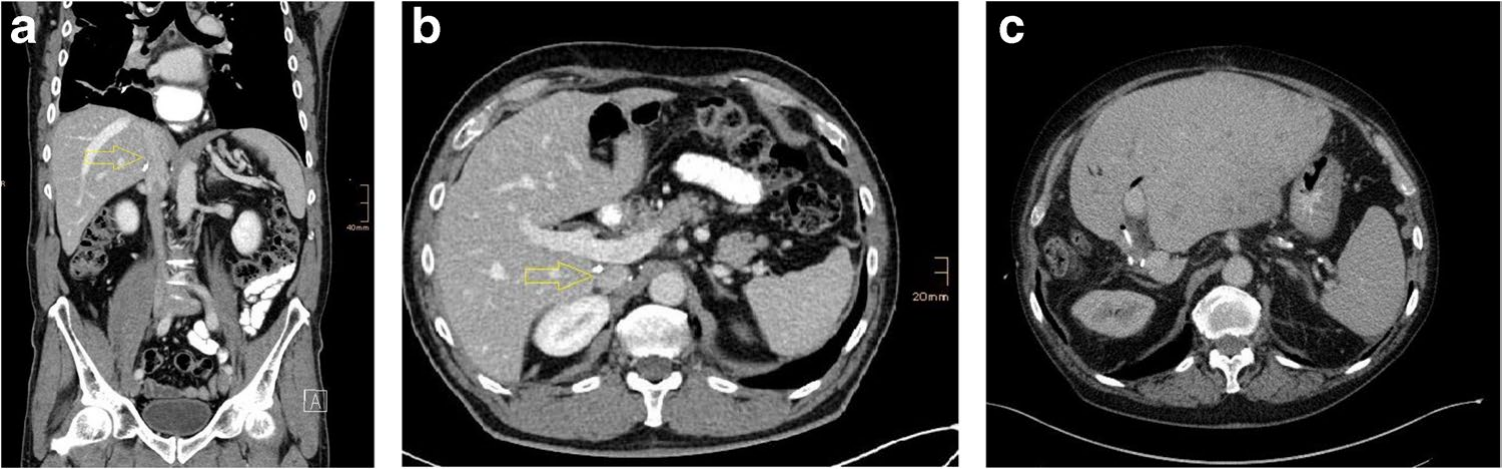
**A** and **B** CT scan of a patient 2 years after right hemihepatectomy with IVC resection and PPP reconstruction for colorectal metastasis without signs of obstruction, stenosis, or thrombosis; **C** CT scan showing good patency of VCI 4 years after reconstruction with PPP during right hemihepatectomy for colorectal metastasis

**Fig. 7:**
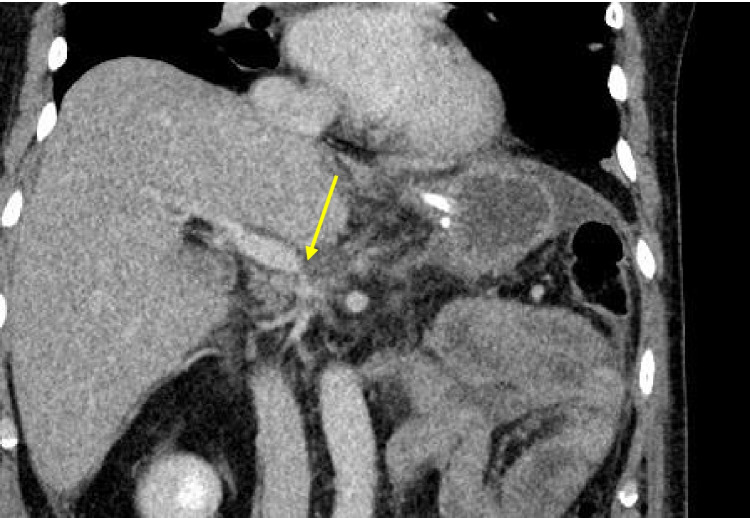
Complications after PPP reconstruction. Stenosis 18 days after PPP reconstruction of PV during pancreatic resection

**Fig. 8:**
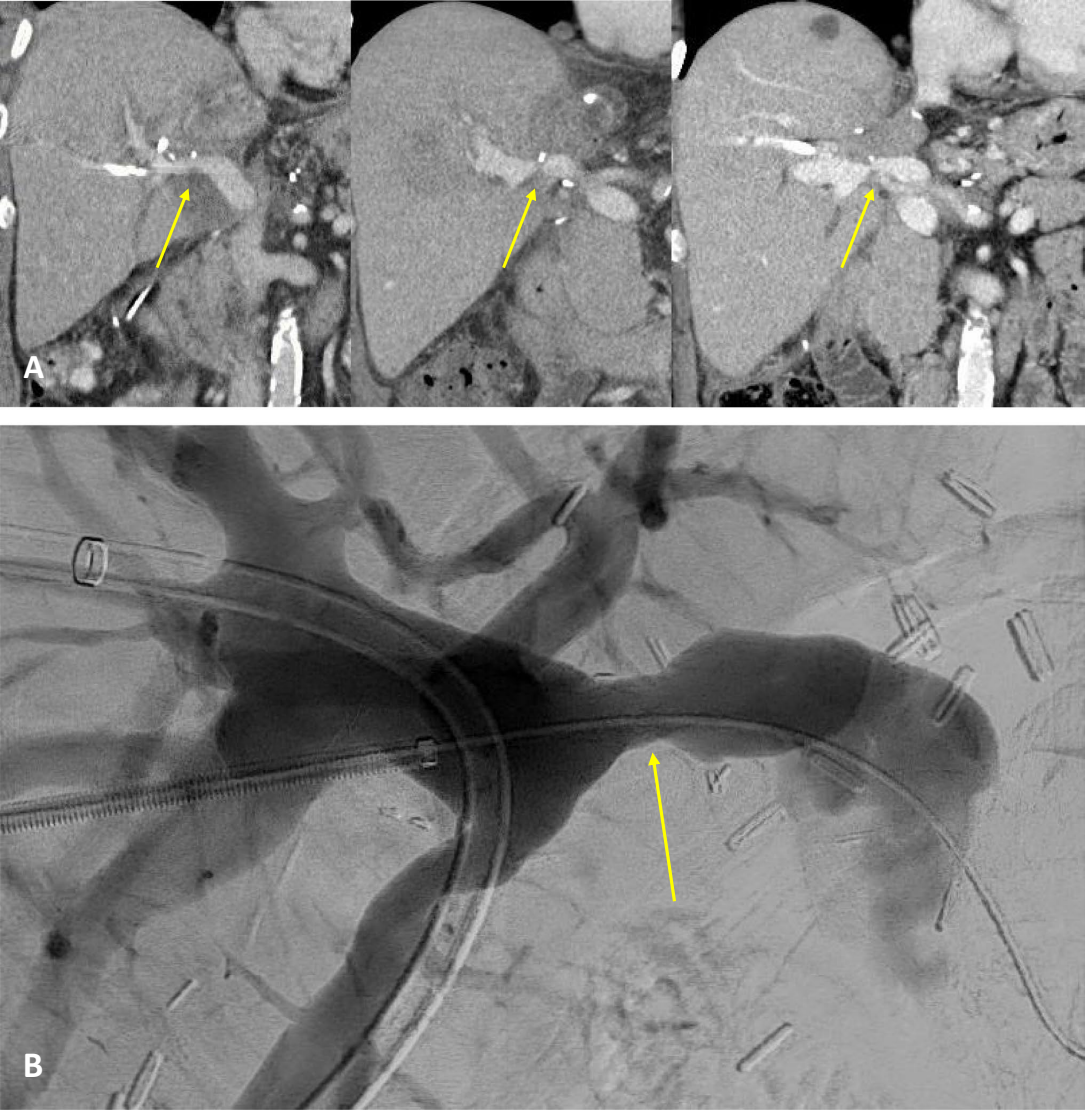
Complications after PPP reconstruction. **A** CT scan of the PV after PPP reconstruction during left hemihepatectomy for CCC with progressive vascular stenosis at 8 days and 2 and 15 months postoperatively; **B** percutaneous transluminal angioplasty (PTA) with stent (arro implementation of PV at 15 months postoperatively)

The overall patency rate in the cohort (*n* = 15: PV *n* = 2; IVC *n* = 13) was 88%. We had two cases of obstruction following a septic postoperative course. In one case after distal pancreatic resection with PPP reconstruction of the portal vein, complete vessel obstruction was found during a complicated postoperative course with a POPF C and sepsis due to necrotizing pancreatitis (Fig. 6). The second case of obstruction occurred after left hemihepatectomy with a common ostium biliodigestive anastomosis to the anterior and posterior pedicle and postoperative anastomotic bile leakage where percutaneous transhepatic cholangial drainage (PTCD) was performed. After initial stenosis 2 months postoperatively, portal hypertension with recurrent gastrointestinal bleeding occurred and CT scans showed high-grade stenosis (more than two-thirds of the lumen) of the portal vein. After 15 months, percutaneous transluminal angioplasty (PTA) and a stent were needed to restore portal blood flow (Figs. 7 and 8).

Although the presence of thrombosis could not be proven by the performed image-based diagnostic in those both cases, we suppose the septic situation to be a possible trigger for the impairment of postoperative patency. Nevertheless, these two patients received therapeutic anticoagulation for treatment.

Apart from these cases, there were no other clinical, paraclinical, or radiological signs of stenosis, obstruction, or thrombosis in the cohort.

## Discussion

Surgical resection with complete tumor clearance improves patient survival and is superior to palliative therapy with regard to long-term outcome in cases with locally advanced tumors [1, 3, 9]. As a result of improved operative techniques, venous resection is a feasible, standard procedure in high-volume centers [10, 11, 12]. The reconstruction techniques follow the principles of vascular surgery. If primary anastomosis is not possible, a tube graft interposition or patch is applied for reconstruction.

### Decision for the type of interponate

The decision for the graft is made depending on the vessel lumen, the type of vascular reconstruction (patch plastic or a tube substitute) and the septic contamination of the surgical procedure, the availability of the graft, and the individual expertise.

In HPB surgery, conventional alloplastic materials such as polyethylene terephthalate (PET) grafts (Dacron®) and (ringed) polytetrafluoroethylene (PTFE) grafts (Gore-Tex®, Gore, USA) are the ones most often used for vascular replacement. In case of high probability for local septic complications such as biliary leakage or pancreatic fistula, autologous material is preferred for the reconstruction. Cryopreserved homografts are mostly used in vascular surgery for reconstruction in the presence of infections for example in abdominal aortic surgery. Data from the literature show comparable rate of graft thrombosis and long-term graft patency for cryopreserved homografts as other biological grafts. However, the need of postoperative immunosuppression and ABO group compatibility is still controversial. We did not use allogenic material since in our collective the reconstruction could be performed, in the majority of cases, with autologous tissue. While autologous venous grafts are suitable and often used, either an additional incision is frequently needed for extraction and/or operation time is longer. In case of a tube graft interposition, we still prefer to harvest an autologous vessel like the saphenous or the jugular vein, which is more suitable for the reconstruction in terms of lumen size as the ovarian or spermatic vein.

For reconstruction with autologous parietal peritoneum, patches can be harvested from the abdominal wall through the same laparotomy without additional incisions. Compared to autologous venous grafts, this technique has several other advantages. The material is easily available, without a considerable increase of the operation time and additional surgical procedure as in case of harvesting another vessel like ovarian/spermatic, saphena, and left renal vein and there is no size limitation. Furthermore, the risk of infection is lower, PPP`s are less expensive and there is no need for postoperative anticoagulation as in the case of synthetic materials [5, 11, 13, 14]. Given all these advantages, vascular reconstruction with peritoneal patches has gained more acceptance in the last decade.

After initial encouraging reports in preclinical studies [15, 16], several case reports followed [16, 17, 18]. The results of a small patient cohort of six patients with PPP reconstruction of the IVC during liver resection were published by the Australian study group of Chin et al. in 1999 [19]. The outcome was encouraging with no mortality or obstruction of the IVC reported postoperatively. The largest series was published in 2015 by the French study group of Dokmak et al. [5]. They reported 52 cases and were the first to use peritoneal patches during pancreatic resection for reconstruction of the portal vein and superior mesenteric vein. Their results showing a patency rate of 97% after application of lateral PPPs are encouraging. The mean follow-up of 14 months for the postoperative CT scan evaluation was nearly comparable to our average time frame of 17 months. However, the majority of the literature still consists of case reports and to the best of our knowledge, there are only four retrospective studies available [6]. In particular, data for pancreatic resections and reconstruction of the portal vein are scarce, which may be one of the reasons for the lack of widespread application yet [20].

In our cohort, PPPs were used in both liver and pancreatic surgery for reconstruction of the IVC and PV. We started using PPPs for IVC reconstruction during paraaortic lymphadenectomy in 2017, with one patient undergoing the procedure. In 2020, we applied this technique in eight patients. Although not included in this paper, we have also used PPPs for reconstruction of the external iliac vein in sarcoma resections with good postoperative results.

Data for the use of PPP for tube graft interposition in literature is even more scarce. A systemic review showed in 2021 three studies with seven interposition of PPP tubes after PV resection. Obstruction was reported in four cases (71%) in the follow-up, and one patient presented with stenosis [6]. These results are somehow disappointing, but the number of patients is limited and therefore not representative. Some better data were obtained on the replacement of the vena cava with PPP tube. In 2013, a study group from Australia performed over 15 tubular interpositions with PPP with no signs of obstruction in the follow-up [21].

Very recent results, reported by the study group of Balzan et al., showed in a small cohort of 8 patients promising results after application of falciform ligament tubular graft for the reconstruction of PV/VMS during Whipple procedure with vascular resection [22]. Seven of the 8 patients showed non-altered vascular patency in CT 6 months postoperatively and a partial thrombosis, clinically inapparent, was diagnosed in one patient. These data are encouraging and the falciform ligament can be used as an alternative to the PPP. However, clinical outcome from prospective studies in larger cohorts of patients is still missing.

### Decision for the side of PPP’s harvesting

PPPs can be harvested from the hypochondrium lateral to the rectus abdominis muscule where peritoneum is slightly thinner compared to the ventral abdominal wall. For the reconstruction of PV, a thinner peritoneum is needed due to its smaller lumen and thinner vessel wall. Some authors prefer even to use the falciforme ligament as a patch or a tubular graft [5, 6]. Due to the small number of patients reported in literature till now, results and long-term outcome for this graft remain unclear [6].

IVC and the hepatic veins (HV) have a thicker and stronger vascular wall and a PPP with a facial layer from the anterior abdominal wall is suitable for the reconstruction. The patency rate reported reaches, although in small patient collectives, up to 100% [5, 19]

### Stenosis and obstruction

Clinical symptoms may indicate the presence of vascular stenosis or obstruction. The symptoms of PV obstruction are initially non-specific as fever, abdominal pain, diarrhea, or ileus and can increase to clinically relevant portal hypertension syndromes presented with ascites and haematemesis. The symptoms of IVC obstruction result from a reduced venous return to the heart. This causes hypotension, tachycardia, edema of the low extremities, elevated liver enzymes, and further organ failure. Diagnostic is accomplished by doppler ultrasound or CT angiography.

According to the French study group, patency rates after PPP reconstruction in liver surgery were better than those observed after pancreatic resection [5]. Our results are in line with these findings. In our small cohort, there were no cases of clinically relevant stenosis postoperatively in patients with reconstruction of the IVC. Two patients developed portal vein obstruction after distal pancreatectomy and hemihepatectomy, respectively (Figs. 5 and 6).

One possible explanation for maintaining postoperative patency after PPP usage could be the inherent fibrinolytic properties of the mesothelium. This aspect was examined in preclinical studies and needs to be studied further [23]. The morbidity rate in our cohort of 24% for patients with CDC ≥ 3b is high but within the reported range for complex multivisceral resections in malignant tumors. Postoperative morbidity was due to sepsis as a result of biliary leakage, pancreatic fistula, or insufficient anastomosis and not related to the patches used.

### Anticoagulation after vascular reconstruction

Although there are no guidelines for the postoperative anticoagulation after venous reconstruction, we defined a standard approach for our institution according to the general guidelines for perioperative venous thromboembolism prophylaxis in general and visceral surgery and the available literature based mainly on case reports [8, 11]. After IVC reconstruction with autologous material, patients receive pharmacological prophylaxis with heparin (low-molecular-weight LMWH or unfractionated UFH) for 4 weeks postoperative if no complication occurs.

After PV reconstruction with autologous graft, prophylactic LMWH doses are applied for a normal diameter ≥ 1.0 cm after reconstruction and half-therapeutic doses of LMWH are applied if the venous diameter was < 1.00 cm.

Our study has some limitations because of its retrospective nature, the heterogeneity of the surgical procedures and the lack of structured follow-up regarding patency rates of vessel reconstructions with PPP. However, it was designed to describe the indications and technical aspects of this still rare procedure. Although data were collected from a small patient cohort, it still represents one of the largest patient cohorts investigated to date. By describing the surgical technique and highlighting important operative steps, we aim to encourage to apply this safe and cost-effective procedure in high-volume centers and further push the borders in surgery of complex multivisceral resections in malignant tumors.

## Conclusions

Our data show promising results for safety and patency rates after PPP for venous reconstruction in liver and pancreatic surgery. In patients undergoing reconstruction of the PV, more studies are needed to evaluate the safety and long-term results of this procedure in this setting.

Moreover, prospective randomized studies should examine the advantage of PPP implantation for vascular reconstruction in both liver and pancreatic surgery.

## Data Availability

Data is available on request from the corresponding author.
